# Microscopic and metatranscriptomic analyses revealed unique cross-domain parasitism between phylum *Candidatus* Patescibacteria/candidate phyla radiation and methanogenic archaea in anaerobic ecosystems

**DOI:** 10.1128/mbio.03102-23

**Published:** 2024-02-07

**Authors:** Kyohei Kuroda, Meri Nakajima, Ryosuke Nakai, Yuga Hirakata, Shuka Kagemasa, Kengo Kubota, Taro Q. P. Noguchi, Kyosuke Yamamoto, Hisashi Satoh, Masaru K. Nobu, Takashi Narihiro

**Affiliations:** 1 Bioproduction Research Institute, National Institute of Advanced Industrial Science and Technology (AIST), Sapporo, Hokkaido, Japan; 2 Division of Environmental Engineering, Faculty of Engineering, Hokkaido University, Hokkaido, Japan; 3 Bioproduction Research Institute, National Institute of Advanced Industrial Science and Technology (AIST), Tsukuba, Ibaraki, Japan; 4 Department of Civil and Environmental Engineering, National Institute of Technology, Anan College, Anan, Tokushima, Japan; 5 Department of Civil and Environmental Engineering, Graduate School of Engineering, Tohoku University, Sendai, Miyagi, Japan; 6 Department of Frontier Sciences for Advanced Environment, Graduate School of Environmental Studies, Tohoku University, Sendai, Miyagi, Japan; 7 Department of Chemical Science and Engineering, National Institute of Technology, Miyakonojo College, Miyakonojo, Miyazaki, Japan; 8 Institute for Extra-cutting-edge Science and Technology Avant-garde Research (X-star), Japan Agency for Marine-Earth Science and Technology (JAMSTEC), Yokosuka, Kanagawa, Japan; Oregon State University, Corvallis, Oregon, USA

**Keywords:** candidate phyla radiation (CPR), *Candidatus *Patescibacteria, cross-domain parasitism, *Candidatus *Yanofskyibacteriaceae, *Candidatus *Minisyncoccaceae, scanning electron microscopy (SEM), transmission electron microscopy (TEM), metatranscriptomic analysis

## Abstract

To verify whether members of the phylum *Candidatus* Patescibacteria parasitize archaea, we applied cultivation, microscopy, metatranscriptomic, and protein structure prediction analyses on the Patescibacteria-enriched cultures derived from a methanogenic bioreactor. Amendment of cultures with exogenous methanogenic archaea, acetate, amino acids, and nucleoside monophosphates increased the relative abundance of *Ca*. Patescibacteria. The predominant *Ca*. Patescibacteria were families *Ca*. Yanofskyibacteriaceae and *Ca*. Minisyncoccaceae, and the former showed positive linear relationships (*r^2^* ≥ 0.70) *Methanothrix* in their relative abundances, suggesting related growth patterns. *Methanothrix* and *Methanospirillum* cells with attached *Ca*. Yanofskyibacteriaceae and *Ca*. Minisyncoccaceae, respectively, had significantly lower cellular activity than those of the methanogens without *Ca*. Patescibacteria, as extrapolated from fluorescence *in situ* hybridization-based fluorescence. We also observed that parasitized methanogens often had cell surface deformations. Some *Methanothrix*-like filamentous cells were dented where the submicron cells were attached. *Ca*. Yanofskyibacteriaceae and *Ca*. Minisyncoccaceae highly expressed extracellular enzymes, and based on structural predictions, some contained peptidoglycan-binding domains with potential involvement in host cell attachment. Collectively, we propose that the interactions of *Ca*. Yanofskyibacteriaceae and *Ca*. Minisyncoccaceae with methanogenic archaea are parasitisms.

**IMPORTANCE:**

Culture-independent DNA sequencing approaches have explored diverse yet-to-be-cultured microorganisms and have significantly expanded the tree of life in recent years. One major lineage of the domain Bacteria, *Ca*. Patescibacteria (also known as candidate phyla radiation), is widely distributed in natural and engineered ecosystems and has been thought to be dependent on host bacteria due to the lack of several biosynthetic pathways and small cell/genome size. Although bacteria-parasitizing or bacteria-preying *Ca*. Patescibacteria have been described, our recent studies revealed that some lineages can specifically interact with archaea. In this study, we provide strong evidence that the relationship is parasitic, shedding light on overlooked roles of *Ca*. Patescibacteria in anaerobic habitats.

## OBSERVATION

Candidate phyla radiation (CPR) or the phylum *Candidatus* Patescibacteria is a lineage of ultrasmall bacteria widely distributed in various natural and artificial environments (1–5). To date, several *Ca*. Patescibacteria-bacteria intradomain parasitism and predatory lifestyles have been observed (e.g., class *Ca*. Saccharimonadia with phylum Actinobacteria (6, 7) and class *Ca*. Gracilibacteria with class Gammaproteobacteria (8, 9), respectively). Recently, cross-domain interactions with archaea have also been discovered for three lineages of the class *Ca*. Paceibacteria (formerly, Parcubacteria/OD1) using cultivation and microscopic observations: *Ca*. Yanofskybacteria/UBA5738 (proposed as family *Ca*. Yanofskyibacteriaceae in this study) (10), *Ca*. Nealsonbacteria (11), and 32-520/UBA5633 (proposed as family *Ca*. Minisyncoccaceae) (12). In all cases, the hosting archaea are methanogens—*Methanothrix* for the former two and *Methanospirillum* for the latter. Host cells with attached *Ca*. Paceibacteria showed markedly low ribosomal activity (based on fluorescence *in situ* hybridization [FISH]) and deformations at the attachment sites (based on transmission electron microscopy [TEM]) (10, 12), suggesting that the *Ca*. Paceibacteria are parasitic. In addition, several genetic features that may contribute to parasitism have been identified in the metagenome-assembled genomes (MAGs) of *Ca*. Paceibacteria (10–12). Here, through successful sustained growth of cultures containing the *Ca*. Paceibacteria and archaea pairs, we were able to couple microscopy and metatranscriptomics to characterize the behavior/mechanisms facilitating the parasitism. Based on our observations, we propose *Ca*. Yanofskyibacterium parasiticum gen. nov. sp. nov. belonging to *Ca*. Yanofskyibacteriaceae, *Ca*. Minisyncoccus archaeophilus gen. nov. sp. nov., and *Ca*. Microsyncoccus archaeolyticus gen. nov. sp. nov. (which belong to *Ca*. Minisyncoccaceae).

To set up the experimental design for these analyses, we prepared seven parallel cultures (termed C-1 to C-7) transferred from the culture C-d2-d1 with high abundances of *Ca*. Patescibacteria described previously (10), which contains acetate, amino acids, and nucleoside monophosphates as potential growth factors for *Ca*. Patescibacteria (see Text S1). The cultures showed production of methane on days 14 and 31. The microbial community structures of the cultures on days 7, 14, 21, and 31 were analyzed using 16S ribosomal RNA gene amplicon sequencing. The abundances of *Ca*. Yanofskyibacterium parasiticum (OTU0011; PMX_810_sub as the corresponding MAG), *Ca*. Minisyncoccus archaeophilus (OTU0014; PMX.108), and *Ca*. Microsyncoccus archaeolyticus (OTU0072; PMX.50) (Fig. S1A and B) during cultivation were, respectively, in the ranges of 0.15%–12.5%, 0.6%–2.3%, and 0.1%–0.87% (Fig. S2A through D; Text S1).

**Fig 1:**
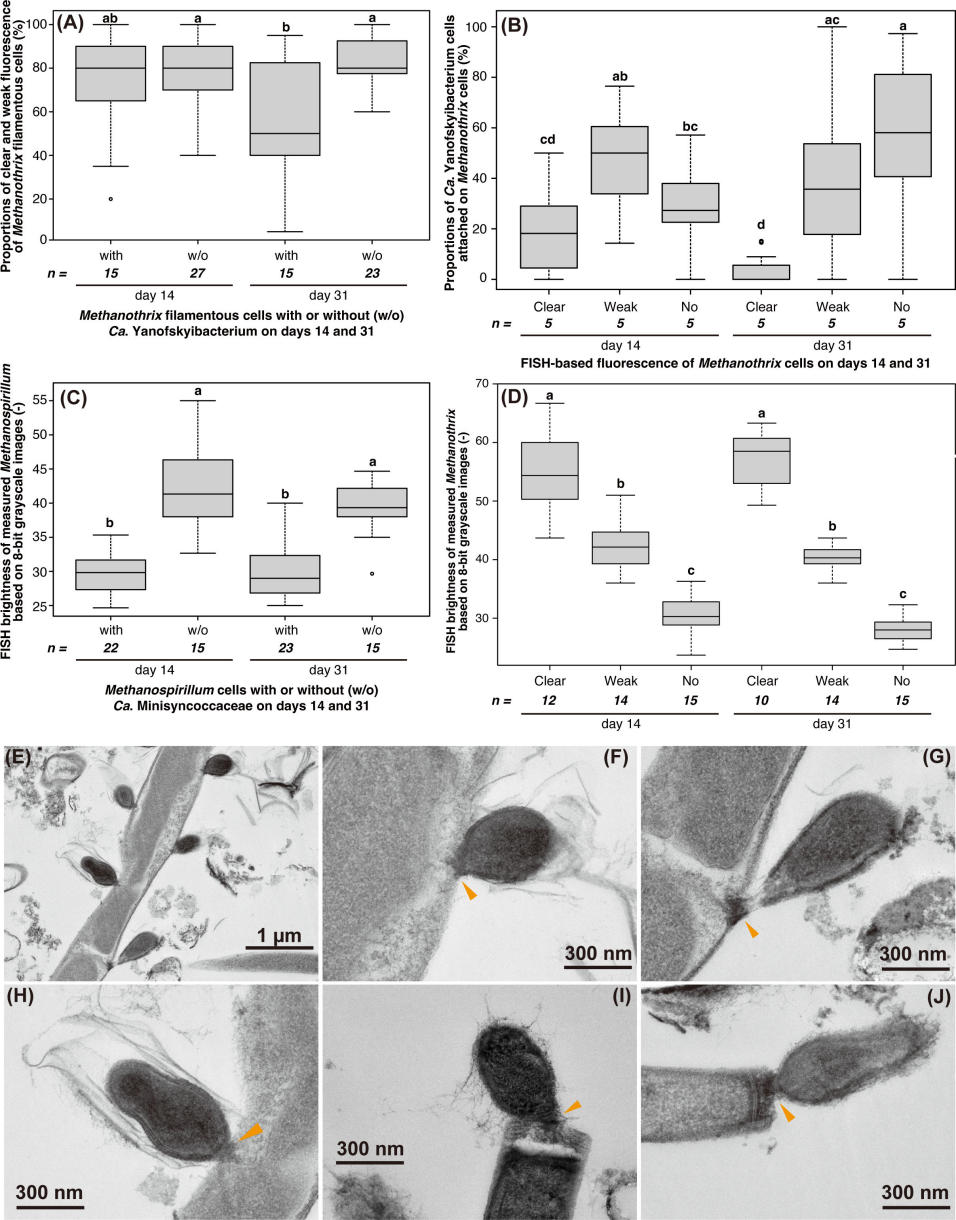
(**A**) Cell length proportions of clear and weak fluorescence of *Methanothrix* filamentous cells calculated based on the fluorescence *in situ* hybridization (FISH) signals using the *Methanothrix*-targeting MX825-FITC probe and *Candidatus* Yanofskyibacterium-targeting Pac_683-Cy3 probe. The *Methanothrix* cells attached with >5 *Ca*. Yanofskyibacterium cells were chosen for calculation. (**B**) Proportions of detected *Ca*. Yanofskyibacterium cells attached to the different fluorescence of *Methanothrix* cells: attached to *Methanothrix* filamentous cells with clear fluorescence, attached to *Methanothrix* with weak fluorescence, and attached to *Methanothrix* with no fluorescence. (**C**) The fluorescence of *Methanospirillum* cells with or without *Ca*. Minisyncoccaceae cells. (**D**) FISH brightness of measured *Methanothrix* filamentous cells based on 8-bit grayscale images. (**A**–**D**) Different letters in the figure indicate significant differences among the values of the proportions based on Tukey’s test (*P* < 0.05). (**E**–**I**) Transmission electron micrographs of small submicron cells attached to (**E**–**H**) *Methanothrix*-like cells and (**I **and **J**) *Methanospirillum*-like cells in culture system C-1 on day 33. Orange arrows indicate extracellular substances at the attachment sites.

**Fig 2:**
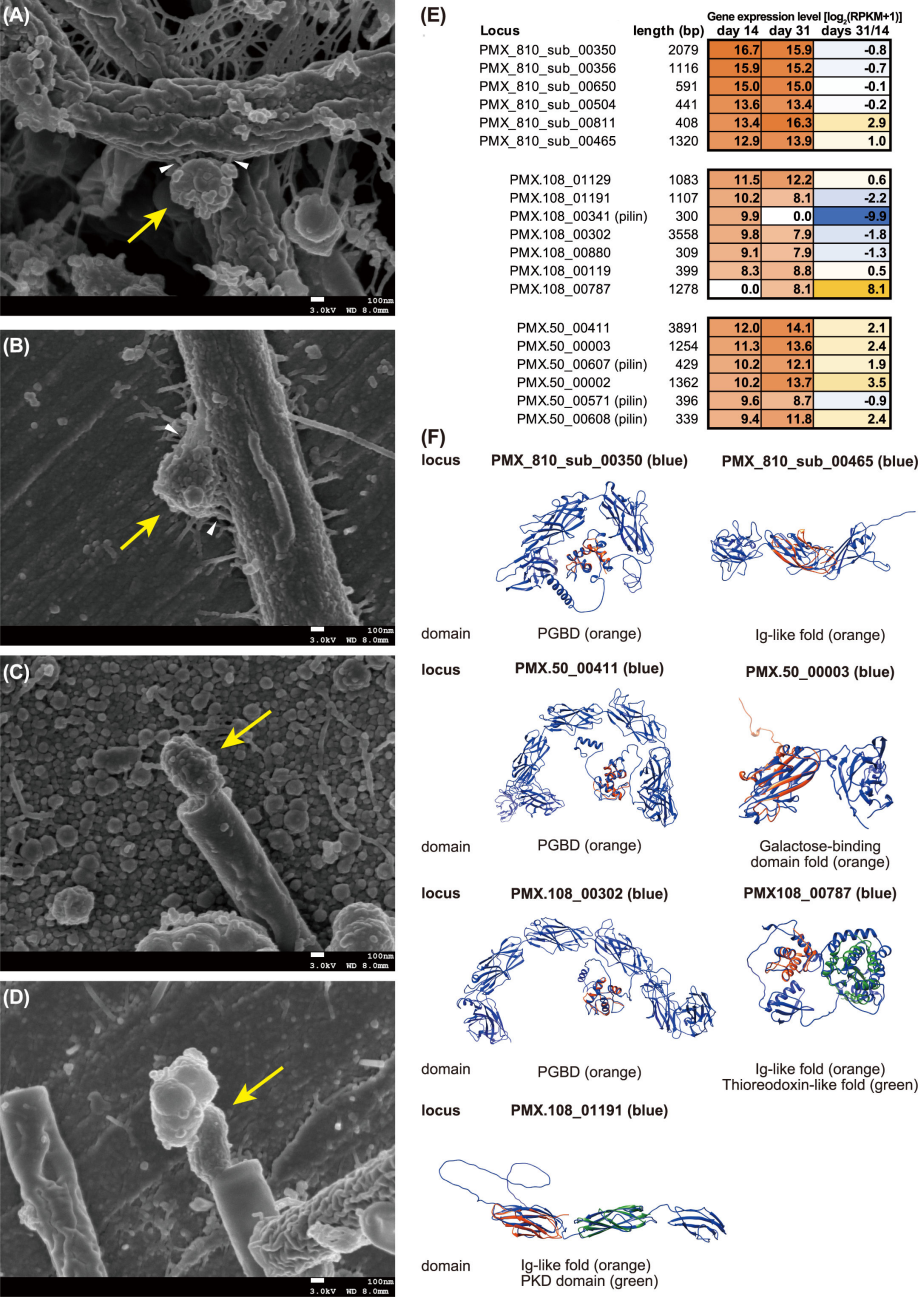
(A–**D**) Scanning electron micrographs of small submicron cells (yellow arrows) attached to (**A** and **B**) *Methanothrix*-like cells and (**C** and **D**) *Methanospirillum*-like cells in culture system C-1 on day 33. White arrows indicate extracellular substances at the attachment sites. (**E**) Gene expression heatmap of the five most highly expressed genes with signal peptides in the genome of *Ca.* Yanofskyibacterium (PMX.810_sub) and *Ca*. Minisyncoccaceae (PMX.50 and PMX.108) in culture systems from C-2 to C-4 on day 14 and C-6 and C-7 on day 31. The color scale from white to orange shows the gene expression level based on the normalized reads per kilobase of transcript per million mapped reads (RPKM) value (see Text S1). “Days 31/14” indicates the difference in gene expression between days 31 and 14. (**F**) Predicted protein structures of the highly expressed genes of *Ca*. Patescibacteria using the AlphaFold2 software package (14). The overlaying domain was predicted through the InterPro database (http://www.ebi.ac.uk/interpro/). PGBD, Ig-like, and PKD are the peptidoglycan-binding domain, immunoglobulin-like domain, and polycystic kidney disease domain, respectively.

The physiological and morphological characteristics of the *Ca*. Paceibacteria–methanogen interactions were confirmed by microscopic observations based on FISH, TEM, and scanning electron microscopy (SEM). On day 31, the FISH fluorescence of *Methanothrix* filaments with more than five *Ca*. Yanofskyibacterium cells was significantly lower than that of *Methanothrix* cells without *Ca*. Yanofskyibacterium cells because of the significantly larger areas with no fluorescence (Fig. 1A; *P* < 0.05). Likewise, comparing *Methanothrix* filaments with different levels of fluorescence showed clear association of the *Ca*. Yanofskyibacterium cells with *Methanothrix* showing no fluorescence (35 ± 25 cells per *Methanothrix* filament) (Fig. 1B and D; Fig. S3A and S4). *Methanospirillum* cells with *Ca*. Minisyncoccaceae cells (from 1.1 ± 0.3 to 1.3 ± 0.5 cells per *Methanospirillum* cell; Fig. S3B) also had significantly lower FISH signals than those without *Ca*. Minisyncoccaceae (Fig. 1C; Fig. S5; *P* < 0.05). Taken together, we conclude that the studied *Ca*. Paceibacteria parasitize methanogenic archaea, strongly supporting previous predictions with statistical evidence (10, 12). As further indirect evidence for parasitism, the cell walls of *Methanothrix* (sheathed filamentous cells) (13) were often deformed where the submicron cells were attached (Fig. 1E through H) in TEM images. We also observed extracellular substances between the submicron cells and hosting *Methanothrix* (Fig. 2A and B) and *Methanospirillum* cells (Fig. 2C and D), suggesting that such materials are important for parasitism by *Ca*. Paceibacteria. High-resolution imaging techniques such as cryo-electron microscopy are necessary to further clarify details of the mechanisms/structures underlying attachment to the host.

To confirm their interactions based on gene expression levels, we performed metatranscriptomics for the enrichment cultures on days 14 (triplicate) and 31 (duplicate). A total of 6.0–10.8 Gb sequences were obtained and mapped to the previously reconstructed Paceibacterales MAGs of *Ca*. Yanofskyibacterium (PMX_810_sub) (10), *Ca*. Microsyncoccus (PMX.50), and *Ca*. Minisyncoccus (PMX.108) (12) (Fig. S1B; Text S1). Previous studies have suggested that the competence protein ComEC, secretion systems, pilus, and several transporters are important for the parasitism or predatory lifestyles of ultrasmall microbes, including *Ca*. Patescibacteria (15–18). Accordingly, these genes were highly expressed in the genome of *Ca*. Yanofskyibacterium PMX_810_sub on day 14 (Table S4). In addition, F-type H^+^-transporting ATPase proteins were highly expressed in *Ca*. Yanofskyibacterium and *Ca*. Microsyncoccus PMX.50 (Tables S2 and S4), which are encoded by type IV pilus assembly proteins (Table S2). In a previous study, ATPase and type IV pili were predicted to function in attachment and motility on larger host surfaces (18). Furthermore, some active peptidase-like proteins with signal peptides (PMX_810_sub_00385, PMX_810_sub_00508, PMX.108_00125, PMX.108_00310, PMX.108_00457, PMX.108_00476, and PMX.50_00413) (Table S2) and substrate-binding proteins of amino acid/metal transport systems were found in the three *Ca*. Patescibacteria genomes (Table S4). Although the detailed functions remain unclear, the addition of external sources of amino acids and trace elements may be key factors for the successful enrichment of *Ca*. Patescibacteria. In the gene expression of the methanogens, there were no significant differences between cultivation days 14 and 31 and different co-existing species (Table S3). Further refinement of the cultures is required to elucidate the details of *Ca*. Patescibacteria parasitism (see the supportlemental material in detail).

We computationally predicted the structures of extracellular enzymes encoded by the five most highly expressed genes with unknown function (Fig. 2F; Table S2). These genes possessed peptidoglycan-binding domains (PMX_810_sub_00350, PMX.50_00411, and PMX.108_00302), immunoglobulin-like folds (PMX_810_sub_00465, PMX108_00787, and PMX.108_01191), galactose-binding domain folds (PMX.50_00003), thioredoxin-like domains (PMX.108_00787), polycystic kidney disease domains (PMX.108_01191), and type IV secretion system pilins (PMX.108_00341, PMX.50_00571, PMX.50_00607, and PMX.50_00608). These domains are known to be host adhesion-related proteins, such as membrane-anchored proteins that bind host the membrane (19–21). Of these, the peptidoglycan-binding domain is found at the N- or C-terminus of several enzymes involved in cell wall degradation (e.g., membrane-bound lytic murein transglycosylase B and zinc-containing D-alanyl-D-alanine-cleaving carboxypeptidase) (22). Interestingly, the enzymes containing peptidoglycan-binding domains showed relatively similar structures among the three *Ca*. Patescibacteria (Fig. 2F), suggesting that these proteins may be important in parasitizing archaeal hosts.

In summary, we found that the interactions between the *Ca*. Patescibacteria class *Ca*. Paceibacteria and methanogenic archaea are parasitisms and uncover overlooked physical and mechanistic details of the interaction through the combination of FISH, TEM, and SEM observations and the first successful gene expression analysis of class *Ca*. Paceibacteria. In addition, we identified highly expressed extracellular enzymes with peptidoglycan-binding domains that have similar structures among the three archaea-parasitizing *Ca*. Paceibacteria. Establishment of purified co-cultures of *Ca*. Paceibacteria and methanogens and further detailed characterization of their cell‒cell interactions are essential to clarify the ecological roles of ultrasmall bacteria on anaerobic ecosystems.

### Description of *Candidatus* Yanofskyibacterium parasiticum sp. nov.

Yanofskyibacterium parasiticum (pa.ra.si.ti.cum. L. neut. adj. parasiticum, parasitic).

Cells are obligate parasitic and coccoid-like and are grown under the anaerobic conditions with the specific host archaeon *Methanothrix* spp. Growth occurs with acetate, various amino acids, and nucleoside monophosphate in a co-culture with the host archaeon. DNA G+C content is 46.7% based on the genomic sequence. The species was obtained from *Candidatus* Patescibacteria-enriched culture from an anaerobic bioreactor-treating purified terephthalate- and dimethyl terephthalate-manufacturing wastewater in Sapporo, Hokkaido, Japan. The species belongs to the genus *Candidatus* Yanofskyibacterium of the family *Candidatus* Yanofskyibacteriaceae. The nearly full-length of 16S rRNA gene sequence of *Candidatus* Yanofskyibacterium parasiticum PMX_810_sub has been deposited in the DDBJ/GenBank/EMBL under the accession number LC715099. The delineation of the species has been proposed by phylogenetic information from genomic sequences. We designate the MAG (BTXX01000000) as the type material of this species.

### Description of *Candidatus* Yanofskyibacterium gen. nov.

Yanofskyibacterium (Ya.nof.sky.i.bac.te’ri.um. N.L. neut. n. bacterium, rod or staff and, in biology, a bacterium; N.L. neut. n. Yanofskyibacterium, named after Charles Yanofsky, who received the ASM Lifetime Achievement Award in 1998).

The genus belongs to the family *Candidatus* Yanofskyibacteriaceae of the order *Candidatus* Paceibacterales. The delineation of the genus has been proposed by phylogenetic information from genomic sequences.

### Description of *Candidatus* Yanofskyibacteriaceae fam. nov.

Yanofskyibacteriaceae (Ya.nof.sky.i.bac.te.ri.a.ce’ae. N.L. neut. n. Yanofskyibacterium, type genus of the family; -aceae, ending to denote a family; N.L. fem. pl. n. Yanofskyibacteriaceae, the family of the genus Yanofskyibacterium).

The family belongs to the order *Candidatus* Paceibacterales of the class *Candidatus* Paceibacteria. The delineation of the family has been proposed by phylogenetic information from genomic sequences.

### Description of *Candidatus* Minisyncoccus archaeophilus sp. nov.

Minisyncoccus archaeophilus (ar.chae.o’phi.lus. Gr. masc. adj. archaîos, ancient; N.L. masc. adj. suff. -philus, friend, loving; N.L. masc. adj. archaeophilus, archaea loving).

Cells are obligate parasitic and are grown under the anaerobic conditions with the specific host archaeon *Methanospirillum* spp. Growth occurs with acetate, various amino acids, and nucleoside monophosphate in a co-culture with the host archaeon. DNA G+C content is 36.4% based on the genomic sequence. The species was obtained from *Candidatus* Patescibacteria-enriched culture from an anaerobic bioreactor treating purified terephthalate- and dimethyl terephthalate-manufacturing wastewater in Sapporo, Hokkaido, Japan. The species belongs to the genus *Candidatus* Minisyncoccus of the family *Candidatus* Minisyncoccaceae. The nearly full-length of 16S rRNA gene sequence of *Candidatus* Minisyncoccus archaeophilus PMX.108 has been deposited in the DDBJ/GenBank/EMBL under the accession number LC715100. The delineation of the species has been proposed by phylogenetic information from genomic sequences. We designate the MAG (BTXZ01000000) as the type material of this species.

### Description of *Candidatus* Minisyncoccus gen. nov.

Minisyncoccus (Mi.ni.syn.coc’cus. L. comp. masc. adj. minor, smaller, inferior; Gr. prep. syn, together; N.L. masc. n. coccus, coccus; from Gr. masc. n. kokkos, grain, seed; N.L. masc. n. Minisyncoccus, small coccus which lives together with another species).

The genus belongs to the family *Candidatus* Minisyncoccaceae of the order *Candidatus* Paceibacterales. The delineation of the genus has been proposed by phylogenetic information from genomic sequences.

### Description of *Candidatus* Microsyncoccus archaeolyticus sp. nov.

Microsyncoccus archaeolyticus (ar.chae.o.ly’ti.cus. Gr. masc. adj. archaîos, ancient; N.L. masc. adj. lyticus, able to loose, able to dissolve; from Gr. masc. adj. lytikos, able to loosen; N.L. masc. adj., archaeolyticus, archaea-dissolving).

Cells are obligate parasitic and are grown under the anaerobic conditions with the specific host archaeon *Methanospirillum* spp. Growth occurs with acetate, various amino acids, and nucleoside monophosphate in a co-culture with the host archaeon. DNA G+C content is 31.1% based on the genomic sequence. The species was obtained from *Candidatus* Patescibacteria-enriched culture from an anaerobic bioreactor treating purified terephthalate- and dimethyl terephthalate-manufacturing wastewater in Sapporo, Hokkaido, Japan. The species belongs to the genus *Candidatus* Microsyncoccus of the family *Candidatus* Minisyncoccaceae. The nearly full-length of 16S rRNA gene sequence of *Candidatus* Microsyncoccus archaeolyticus PMX.50 has been deposited in the DDBJ/GenBank/EMBL under the accession number LC715109. The delineation of the species has been proposed by phylogenetic information from genomic sequences. We designate the MAG (BTXY01000000) as the type material of this species.

### Description of *Candidatus* Microsyncoccus gen. nov.

Microsyncoccus (Mi.cro.syn.coc’cus. Gr. masc. adj. mikros, small, little; Gr. prep. syn, together; N.L. masc. n. coccus, coccus; from Gr. masc. n. kokkos, grain, seed; N.L. masc. n. Microsyncoccus, small coccus which lives together with another species).

The genus belongs to the family *Candidatus* Minisyncoccaceae of the order *Candidatus* Paceibacterales. The delineation of the genus has been proposed by phylogenetic information from genomic sequences of the MAG. The delineation of the genus has been proposed by phylogenetic information from genomic sequences.

### Description of *Candidatus* Minisyncoccaceae fam. nov.

Minisyncoccaceae (Mi.ni.syn.coc.ca.ce’ae. N.L. masc. n. Minisyncoccus, type genus of the family; -aceae, ending to denote a family; N.L. fem. pl. n. Minisyncoccaceae, the family of the genera Minisyncoccus).

The family belongs to the order *Candidatus* Paceibacterales of the class *Candidatus* Paceibacteria. The delineation of the family has been proposed by phylogenetic information from genomic sequences.

## SUPPLEMENTAL MATERIAL

Text S1 mbio.03102-23-s0001.pdf Supplemental text and legends for the supplemental figures and tables.

Fig. S1 mbio.03102-23-s0002.jpg Phylogenetic trees of order *Ca*. Paceibacterales.

Fig. S2 mbio.03102-23-s0003.jpg Relative abundance of predominant *Candidatus* Patescibacteria and methanogenic archaea.

Fig. S3 mbio.03102-23-s0004.jpg Number of *Ca*. Paceibacterales cells attached to methanogenic archaea.

Fig. S4 mbio.03102-23-s0005.jpg Micrographs of *Candidatus* Yanofskyibacterium and Methanothrix.

Fig. S5 mbio.03102-23-s0006.jpg Micrographs of *Candidatus* Minisyncoccaceae and Methanospirillum.

Table S1 mbio.03102-23-s0007.xlsx Mapped and total sequence reads of the metatranscriptome in this study.

Table S2 mbio.03102-23-s0008.xlsx Summary of the gene expression level and annotation of metagenomic bins of *Ca*. Paceibacterales.

Table S3 mbio.03102-23-s0009.xlsx Summary of the gene expression level and annotation of metagenomic bins of methanogenic archaea.

Table S4 mbio.03102-23-s0010.xlsx Summary of the annotation of parasitism-related proteins in the genomes of *Ca*. Paceibacterales.

ASM does not own the copyrights to Supplemental Material that may be linked to, or accessed through, an article. The authors have granted ASM a non-exclusive, world-wide license to publish the Supplemental Material files. Please contact the corresponding author directly for reuse.
