# Supplementary figures and images for: Microscopic and metatranscriptomic analyses revealed unique cross-domain parasitism between phylum Candidatus Patescibacteria/candidate phyla radiation and methanogenic archaea in anaerobic ecosystems
Source: mBio. 2024 Feb 7;15(3):e03102-23. doi: 10.1128/mbio.03102-23 (PMC10936435; doi:10.1128/mbio.03102-23)

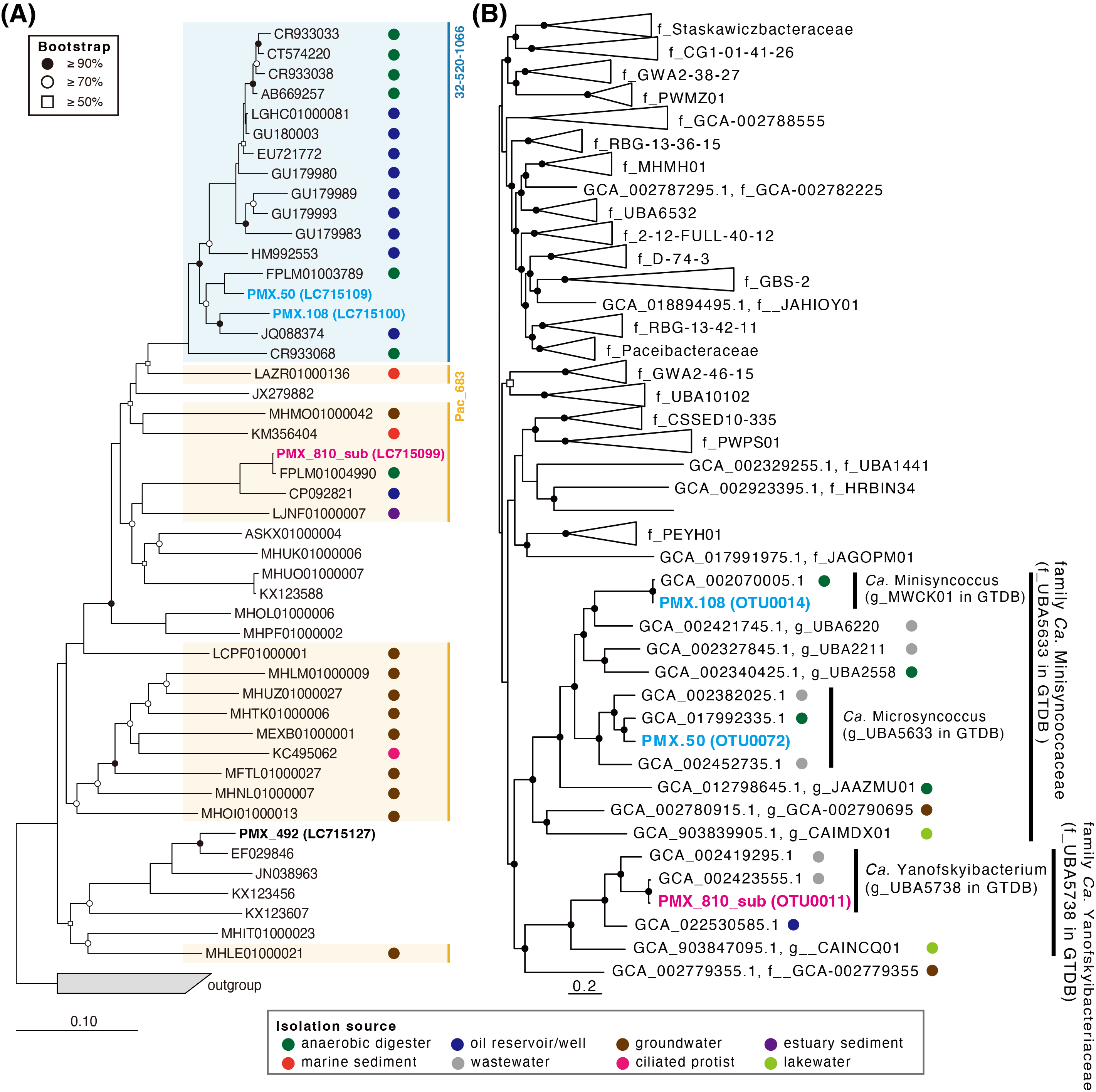

Supplement: Fig. S1 — Phylogenetic trees of order Ca. Paceibacterales. [file mbio.03102-23-s0002.jpg]

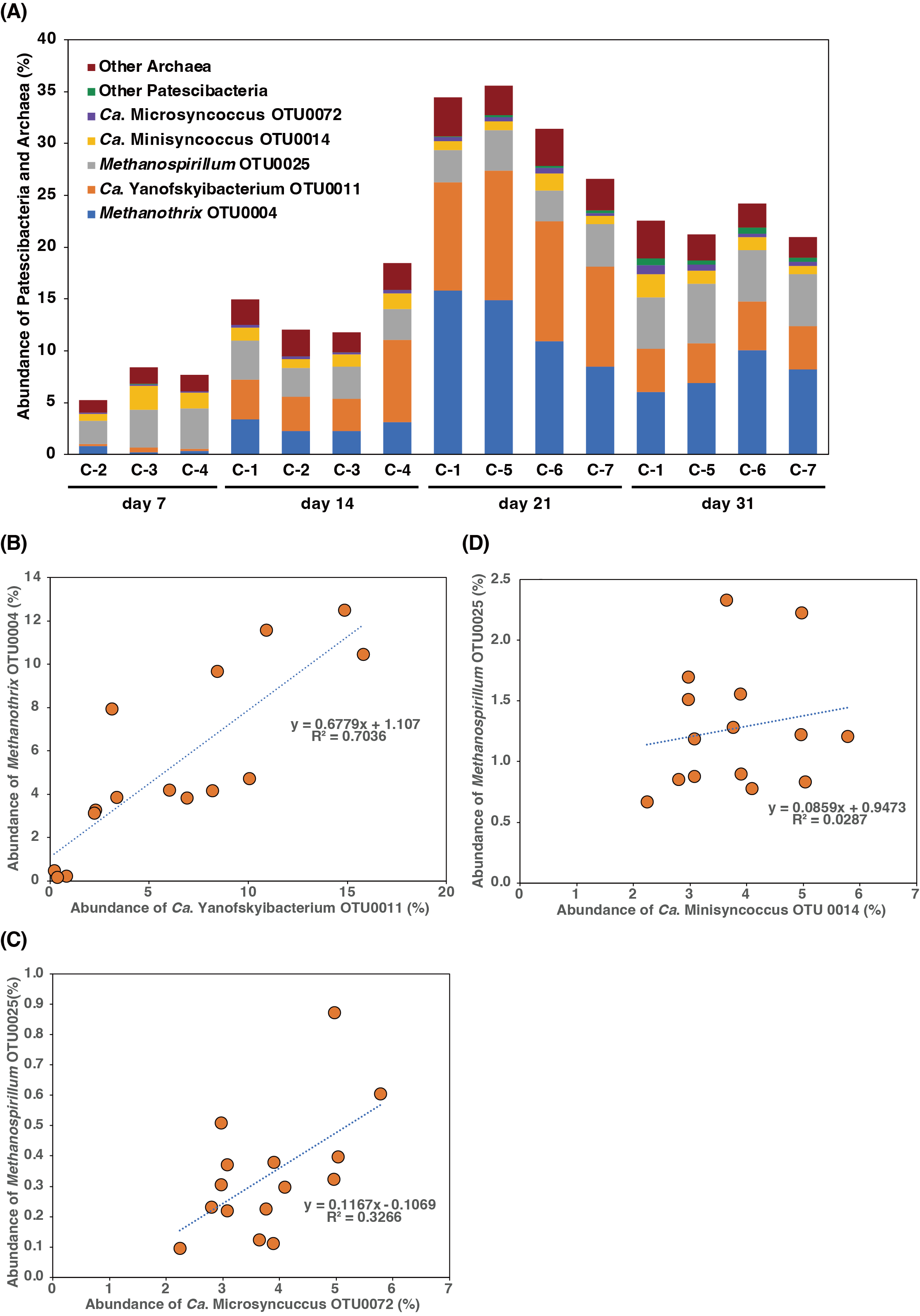

Supplement: Fig. S2 — Relative abundance of predominant Candidatus Patescibacteria and methanogenic archaea. [file mbio.03102-23-s0003.jpg]

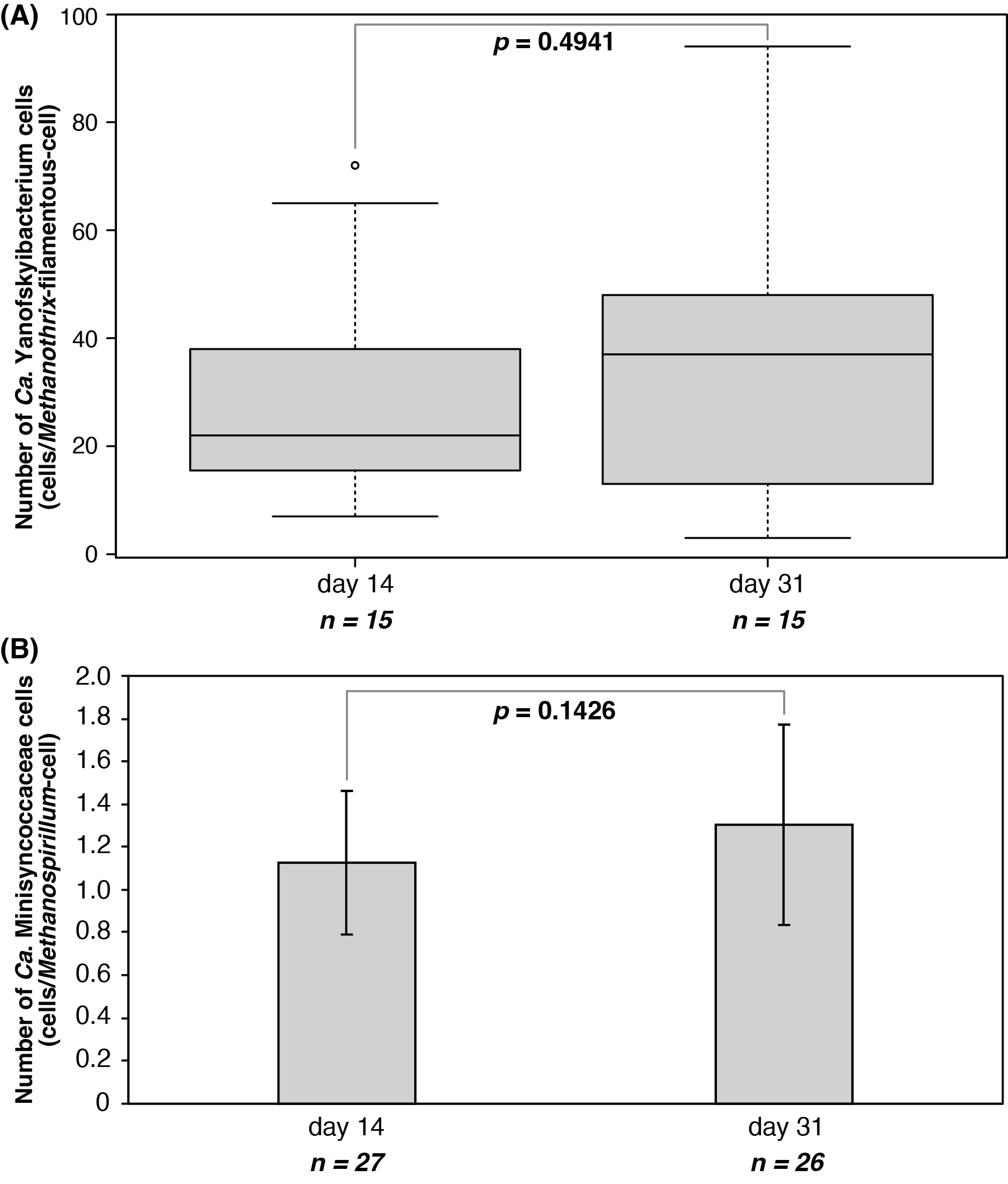

Supplement: Fig. S3 — Number of Ca. Paceibacterales cells attached to methanogenic archaea. [file mbio.03102-23-s0004.jpg]

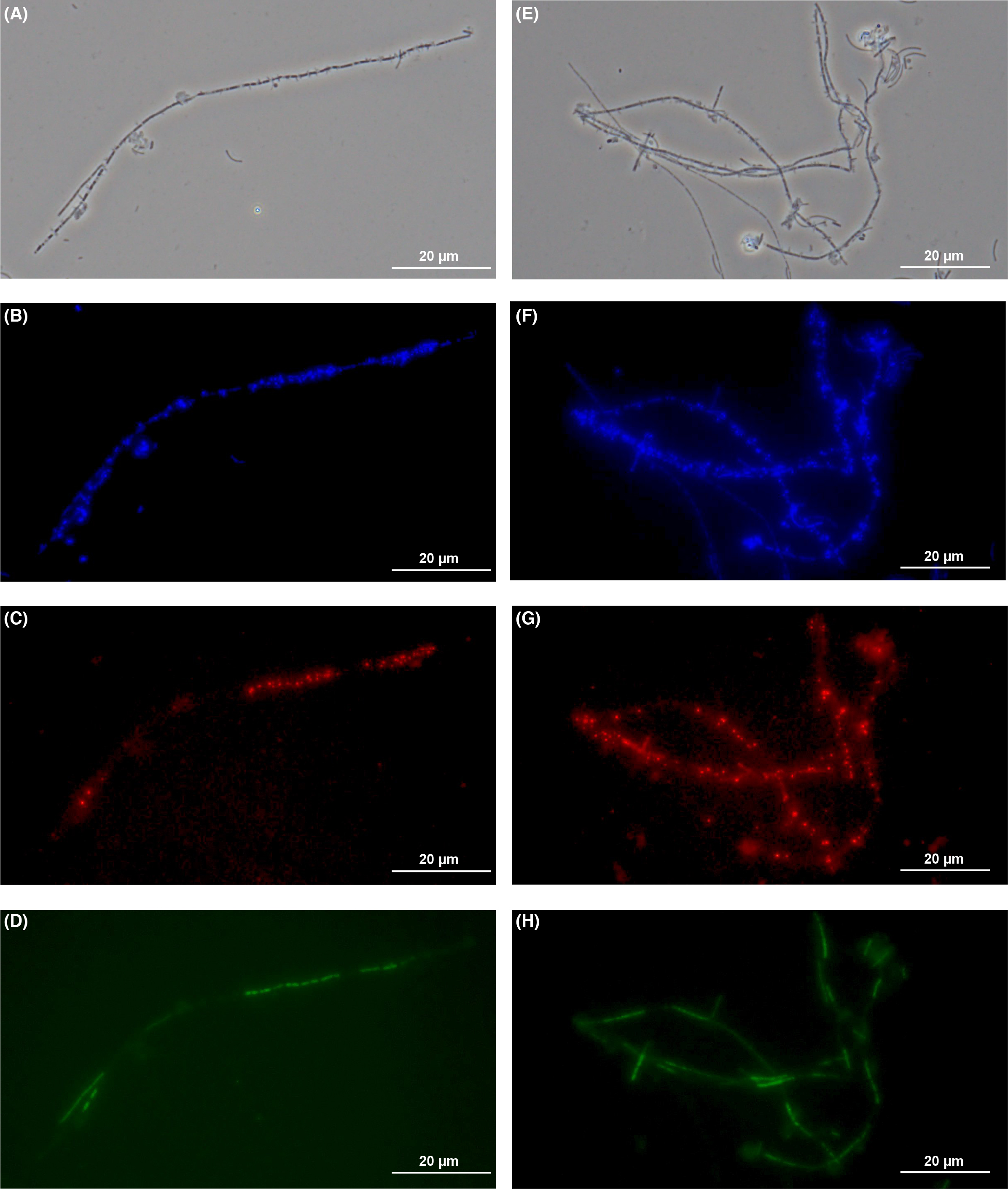

Supplement: Fig. S4 — Micrographs of Candidatus Yanofskyibacterium and Methanothrix. [file mbio.03102-23-s0005.jpg]

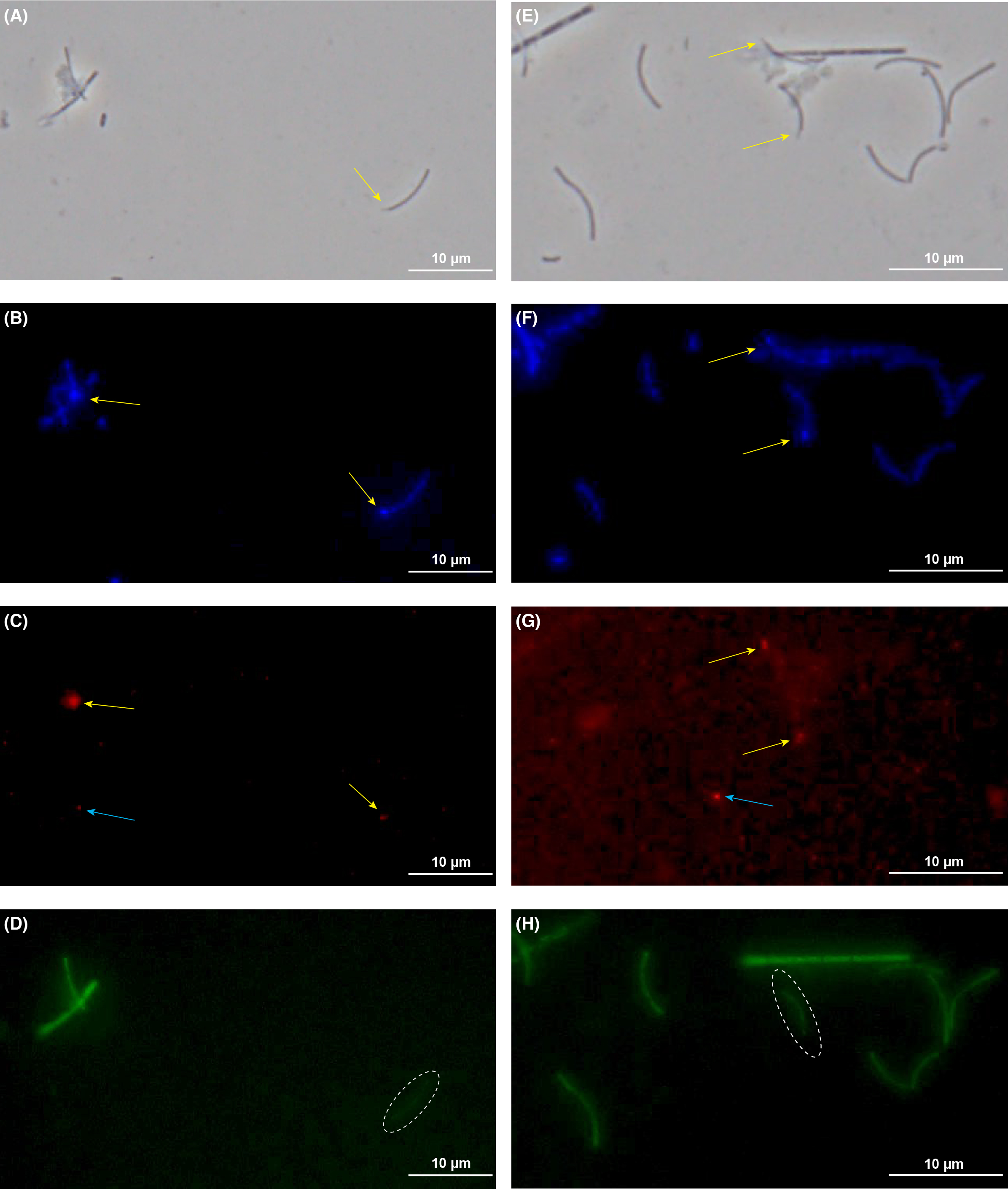

Supplement: Fig. S5 — Micrographs of Candidatus Minisyncoccaceae and Methanospirillum. [file mbio.03102-23-s0006.jpg]
